# An interoceptive model of energy allostasis linking metabolic and mental health

**DOI:** 10.1126/sciadv.ady4356

**Published:** 2025-09-24

**Authors:** Sara Z. Mehrhof, Hugo Fleming, Camilla L. Nord

**Affiliations:** ^1^ MRC Cognition and Brain Sciences Unit, University of Cambridge, Cambridge, UK.; ^2^ Department of Psychiatry, University of Cambridge, Cambridge, UK.

## Abstract

Mental health conditions like depression are associated with an elevated risk of cardiometabolic disorders, yet the mechanisms underlying this comorbidity remain poorly understood. Is metabolic dysfunction a cause of depression, a downstream consequence, or do both stem from shared underlying processes? We argue that neurocognitive mechanisms, particularly those involved in reward and effort processing, interact with metabolic physiology to shape each of these causal pathways. Metabolic signals do not act on the brain in isolation; they are embedded within a broader interoceptive system through which the brain detects and interprets bodily states. This system supports allostasis, the brain’s predictive regulation of internal physiological demands. We propose a framework of interoceptive energy allostasis in which disruptions to these predictive processes contribute to the bidirectional relationship between depression and metabolic dysfunction. By integrating perspectives from metabolic and computational psychiatry, this framework offers a theoretical lens to explain the multidirectional comorbidity between mental and metabolic ill-health.

## Teaser

Interactions between metabolic interoception and regulation may drive comorbidity between mental and metabolic ill-health.

## INTRODUCTION

Mental health conditions reduce life expectancy by an average of 10 to 15 years (*1*). Annually, 8 million deaths worldwide are attributable to mental health conditions, the majority due not to elevated risk for suicide or accidents but to cardiometabolic disease (*2*, *3*). Poor metabolic health (including abdominal obesity, elevated blood glucose, and hypertension) precipitates cardiometabolic conditions and is more prevalent in psychiatric disorders than the general population (*4*). The urgency of closing the “mortality gap” in mental health conditions demands a deeper understanding of the link between metabolic ill-health and psychiatric disorders. Most critically, the nature of their relationship is unclear: Does poor metabolic health increase the risk of mental illness, does mental illness lead to metabolic dysfunction, or do both emerge from shared underlying mechanisms? And in any case: Through what pathways?

Our review focuses particularly on the most common mental health disorder, major depressive disorder. Metabolic risk factors are significantly elevated in depression (*4*), and the risk for type 2 diabetes is increased by more than 50% (*5*). Conversely, an estimated one in four patients with type 2 diabetes experience depression, double the incidence rate of the general population (*6*, *7*). However, major depressive disorder is a heterogeneous diagnosis (*8*), and the association between metabolic health and depression may be driven by particular subsets of symptoms, which may themselves be transdiagnostic in nature. Studies have consistently implicated symptom profiles related to energy regulation and motivation—hyperphagia, weight gain, hypersomnia, and leaden paralysis, as well as anhedonia—with markers of poorer metabolic health in major depression (*9*–*11*). Shared symptom profiles might also explain why metabolic ill-health is common across psychiatric disorders. Nevertheless, a transdiagnostic perspective linking symptom profiles to metabolic ill-health does not clarify the direction of causality or the relevant mechanisms driving the link. This impedes the development of interventions aimed at reducing metabolic risk in these symptom clusters or integrated treatment approaches that target metabolic and mental health problems.

Causal explanations frequently cite lifestyle differences (*12*), metabolic side effects of psychiatric medication [particularly antipsychotic medication and some antidepressants, see (*13*, *14*)], and healthcare inequalities (*12*) as mechanisms for the comorbidity been psychiatric and metabolic ill-health. However, the relationship between the two persists even in populations of treatment-naïve patients (*15*–*17*) and when controlling for lifestyle-related confounders (*5*, *10*, *18*), suggesting neither treatments nor lifestyle differences are likely to fully account for the association. Instead, a growing body of evidence suggests that there are more direct interactions between depression and metabolic ill-health that could explain their comorbidity: Biological processes by which poor metabolic health could contribute to brain processes, known neurocognitive differences in depression that could contribute to metabolic dysfunction, and factors that could precede and influence development of both conditions.

We first argue that changes to neurocognitive mechanisms provide a key missing variable to explain the comorbidity between depression and metabolic ill-health. Biological factors such as insulin signaling exert effects on depression-related neurocognitive systems, providing support for a causal link from metabolic to mental health. Likewise, cognitive features of depression change metabolically relevant behaviors, such as exercise habits. Additionally or alternatively, cognitive antecedents such as motivational differences might confer common risk for both classes of disorders. We review evidence indicating that neurocognitive mechanisms play a central role in driving multidirectional relationships between mental health and metabolic energy regulation. Considering neurocognitive processes as central drivers in the relationship between mental and metabolic health may help disentangle drivers of comorbidity and also identify new possible treatment targets and intervention approaches.

But metabolic signals do not exert direct effects on the brain in isolation. Metabolic signals form part of our interoceptive milieu: a vast array of information about the internal condition of the body, sensed by the brain (*19*). Sensing internal signals, interoception, is known to be disrupted transdiagnostically across psychiatric disorders, including depression (*20*, *21*). Interoceptive signals are thought to be processed predictively, via “allostasis,” enabling the brain to anticipate and resolve potential future disruptions to physiological states and facilitating survival (*22*, *23*).

We propose that the brain predicts and regulates energy balance based on internal bodily signals: a theoretical model of interoceptive energy allostasis that explains the interrelationship between mental and metabolic health. Changes in our metabolic health have the capacity to disrupt this allostatic process, as do higher-order changes in cognitive and neural systems involved in the production or updating of allostatic predictions. Through this model, we suggest that the comorbidity between mental and metabolic ill-health could arise via insults at various levels of our interoceptive allostatic system: for instance, disruptions in metabolic signaling due to dietary changes or sleep-wake cycle disturbances that alter interoceptive energy allostasis from the “bottom up,” or cognitive features of depression that alter energy-relevant learning and decision-making. By integrating perspectives from metabolic psychiatry and computational-cognitive psychiatry, our model begins to explain the complex relationship between depression, energy regulation, and metabolic dysfunction. Our proposed framework should be seen in the light of a rich literature on theories of allostasis, interoception, and active inference and their role in mental and physical health (*22*–*29*). Our framework shares key principles with the hierarchical Bayesian accounts of interoception proposed by Petzschner *et al*. (*24*). However, it differs in scope and emphasis: We extend principles into the metabolic domain, focusing on energy balance and its disruption as a core mechanism linking interoceptive inference to affective symptoms. Our model is deliberately less formal, aiming instead to provide a cross-disciplinary scaffold for understanding and intervening in the comorbidity between depression and metabolic ill-health. We hope that this framework paves the way for better understanding of mental-metabolic comorbidity and illuminates novel areas for future intervention or prevention.

## FROM METABOLIC TO MENTAL HEALTH, AND BACK AGAIN

Across the following three sections, we review evidence for direct pathways between depression and metabolic ill-health, focusing on the role of neurocognition as a linking mechanism. We start by discussing the causal route from metabolic disruption to depression (e.g., disruptions in insulin signaling altering mental health), before exploring reverse causality (e.g., depression increases behavior that confers risk for metabolic ill-health). Last, we discuss putative common risk underlying mental and metabolic comorbidity. Common across all three pathways are parallel changes in neurocognitive mechanisms, including reward processing and decision-making. Our review proposes that these cognitive commonalities could support the notion of multiple points of entry to metabolic-mental health dysfunction.

### The causal route from metabolic to mental health

There are multiple mechanisms by which metabolic disease might cause depression-related changes. In the current text, we specifically focus on two candidate routes: (i) disruption to the neuroendocrine systems, including disruptions to insulin, leptin, glucagon-like peptide-1 (GLP-1), and ghrelin; and (ii) systemic inflammation resulting from metabolic disease. These two pathways are not distinct but rather have overlapping and potentially synergistic effects.

#### Insulin

Perhaps the best example of a neuroendocrine disruption investigated in the context of metabolic-mental health overlap is insulin, a peptide hormone released by the beta cells of the pancreas. Its primary role in the periphery is to regulate blood glucose levels through two main routes: by decreasing endogenous glucose production; and by increasing glucose uptake by muscle, liver, and adipocytes (*30*). Reduced sensitivity of insulin receptors is one of the primary characteristics of type 2 diabetes and causes decreased responsiveness to changes in blood glucose levels, as well as increased fasting blood sugar levels overall (*31*). But in addition to its peripheral roles, insulin readily crosses the blood-brain barrier (*32*) and acts on widely distributed receptors throughout the brain, including the prefrontal cortex, hippocampus, and cerebellum, as well as the ventral tegmental area (VTA) and ventral striatum—key regions of the mesolimbic dopamine pathway involved in reward and motivation (*33*, *34*).

Insulin’s effects in the mesolimbic system are complex and regionally specific [for detailed reviews, see (*35*, *36*)]. In the VTA, insulin suppresses dopaminergic neuron activity, reducing tonic dopamine levels and dampening reward sensitivity [e.g., (*37*); see (*38*) for a detailed review]. This corresponds to behavioral outcomes such as impaired conditioned place preference and elevated thresholds for self-stimulation (*39*, *40*). In contrast, in the ventral striatum, insulin enhances dopamine release, supporting reward learning but also contributing to satiety (*41*–*43*).

Notably, insulin signaling may be critical for mood regulation. In rodents, knockout of insulin receptors reduces striatal dopamine and induces depression-like behavior (*41*, *44*). Together, these findings position central insulin resistance as one route by which metabolic ill-health could lead to depression, via reward processing changes.

This notion is supported by the (rare) human studies in this field: Intranasal (i.e., central) insulin administration engages a characteristic network of brain regions including the VTA and ventral striatum (*45*, *46*) and lowers baseline dopamine levels in the nucleus accumbens (NAc) (*45*, *47*), effects that are blunted or absent in individuals with obesity or peripheral insulin resistance. This suggests that metabolic ill-health may contribute to central insulin resistance (*46*, *48*, *49*), disrupting reward processing and heightening depression risk. Obesity and even a short-term high-calorie diet are linked to impaired reward sensitivity and learning (*50*, *51*). Insulin resistance also varies with depression severity, increasing in acute episodes and diminishing again during remission (*10*). Together, this underscores the potential role of insulin sensitivity in the pathophysiology of depression. Insulin is only one of several neuroendocrine disruptions implicated in mental health; however, below, we outline three others (leptin, ghrelin, and GLP-1), likewise, with the capacity to causally alter neural and cognitive systems supporting mental health.

#### Leptin

Leptin, a hormone typically secreted by white adipose cells, has similar effects on reward and motivation systems. In rodent studies, leptin infused into VTA reduces both tonic and cue-evoked dopamine release, dampening food reward value and effort-related responding (*52*, *53*). Moreover, intracerebroventricular injections of leptin can alter the rewarding effect of brain stimulation (*54*), as well as reduce the rewarding effect of exercise (*55*). Striatal leptin, in contrast, increases extracellular dopamine (*56*). In circulation, leptin concentration communicates the amount of energy now stored as fat in the body (*57*, *58*) and, thus, performs an analogous role to insulin, coordinating energy intake and expenditure by promoting satiety and reduced food-seeking (*59*, *60*).

Knockout of leptin signaling (or impaired receptor function secondary to obesity) in rodents induces depression-like behavior (*61*), while central or peripheral leptin administration has antidepressant effects (*62*, *63*). Perhaps unexpectedly, in humans, elevated serum leptin predicts greater risk of atypical depression, especially in individuals with high abdominal adiposity (*64*, *65*); this may, however, be caused by leptin resistance, which induces a compensatory increase in circulating leptin (*66*). Diminished leptin signaling may, therefore, contribute to both metabolic and affective symptoms of atypical depression, a second metabolic signal changing general neurocognitive factors (reward and motivation) in parallel with its role on appetite-related processes.

#### GLP-1

A smaller number of studies suggest an additional role for GLP-1, a member of the incretin family of gut-derived hormones, which regulate blood glucose primarily by enhancing insulin secretion after food intake. GLP-1 also acts independently, including in the brain, where GLP-1 receptors are expressed throughout the mesolimbic reward system and are colocalized with dopamine receptors (*67*, *68*). In rodents, GLP-1 receptor activation in the VTA and NAc suppresses dopamine release triggered by drug consumption (e.g., amphetamine, cocaine, or alcohol) without altering baseline dopamine levels (*69*, *70*). This reduces drug-induced place preference (*71*, *72*), suggesting a broader role in modulating reward sensitivity or learning. Chronic GLP-1 administration also produces antidepressant-like effects in rodents (*73*).

Despite growing clinical use of GLP-1 agonists for weight loss, few studies have so far examined their effects on reward processing in humans, and results remain inconclusive (*74*). Notable exceptions include studies by Hanssen and colleagues, who found that the GLP-1 agonist liraglutide increased learning rates on an associative learning task (*75*) and restored the motivating effect of hunger in participants with insulin resistance (*76*). Evidence for an association of GLP-1 with depression symptoms is similarly ambivalent according to a recent review (*77*), in part, because these authors were only able to identify four papers on which to report. Nevertheless, the involvement of GLP-1 in reward learning suggests a potential involvement in mental health–related processes and, particularly given the increasing use of GLP-1 agonists, is an important area of future study.

While the current review focuses on depression, it is worth mentioning emerging evidence linking GLP-1 and GLP-1–based treatments to addiction, mediated by its effect on dopaminergic signaling (*78*, *79*). Preclinical studies (*80*), as well as human observational studies (*81*), and emerging randomized controlled trials (*82*) indicate that GLP-1 agonists may reduce the rewarding effects of different addictive substances.

#### Ghrelin

Our final illustrative example of a neuroendocrine disruption with mental health–related consequences is ghrelin. Ghrelin, unlike the other hormones discussed so far, can crudely be thought of as signaling lower energy availability. Released during fasting, it promotes appetite and food intake (*83*). Ghrelin levels are typically reduced in obesity, type 2 diabetes, and metabolic syndrome and may enhance dopaminergic signaling and reward responses (*84*).

The ghrelin receptor is expressed in the VTA, substantia nigra, and ventral striatum (*85*). In rodents, VTA-administered ghrelin increases the firing rate of dopaminergic neurons and boosts both tonic and (food and drug) evoked striatal dopamine (*85*, *86*), as well as motivation to work for food rewards (*85*, *87*). Systemic ghrelin administration also has antidepressant-like effects, for instance, on the forced swim and sucrose-preference tests [see (*88*) for a review], although note that one study has reported opposite effects (*89*).

In humans, findings are mixed: Some report lower baseline ghrelin levels in depression (*90*), while others found no difference between cases and controls (*91*). More recently, a study found that ghrelin levels were increased in patients with severe, although not moderate, depression symptoms (*92*). Epidemiological studies suggest that ghrelin levels predict depression onset over 3 years (*93*) [see also (*88*) for a review], and successful response to antidepressant treatment is associated with a reduction in ghrelin levels (*94*).

In summary, insulin, leptin, GLP-1, and ghrelin all have substantial effects on behavior and cognition through their effects on mesolimbic dopamine pathways. These pathways link reward and motivation to current estimates of energy status, offering a plausible mechanism by which metabolic health impacts cognition and contributes to mental health conditions like depression. Future studies will be needed directly test this hypothesis. For instance, longitudinal studies could track the development of neurocognitive changes and psychiatric symptoms as individuals with metabolic conditions (e.g., insulin resistance) undergo metabolic treatment. We would hypothesize that normalization of neurocognitive processes would precede alleviation in mental health symptoms. A more causal test would involve experimental induction of some aspect of metabolic ill-health (in a randomized, double-blind design) and subsequent monitoring of cognitive changes and psychiatric symptoms development. However, both ethical and practical (lack of suitable intervention) considerations render this direct causal test more difficult.

#### An alternative route from metabolic to mental health: inflammation

Systemic inflammation is an alternative causal influence linking metabolic dysfunction to depression. Inflammatory markers are elevated in individuals with depression (*95*) and predict future depression risk over 4 years (*96*). Inflammation is especially pronounced in atypical depression, which is also characterized by increased adiposity and metabolic dysfunction (*97*). Metabolic ill-health increases peripheral inflammation via cytokine release from white adipocytes, potentially also promoting neuroinflammation (*65*). This suggests inflammation may partly mediate the link between type 2 diabetes and depression (*38*, *98*), particularly through increasing anhedonia, which is both correlated with inflammatory markers (*99*, *100*) and responsive to anti-inflammatory treatments (*101*).

As with endocrine disruption, inflammation alters reward processing circuits. Specifically, inflammation is associated with attenuated reward-related activity in prefrontal and striatal regions (*102*, *103*), potentially owing to decreased striatal dopamine (*104*, *105*). Inducing inflammation experimentally leads to reduced effort for reward (*106*, *107*), while anti-inflammatory treatment improves willingness to exert effort in depressed patients with high inflammatory markers (*108*). In short, inflammation reduces motivation, possibly to conserve energy due to increased energy demands by the immune system (*109*).

Inflammation, therefore, represents a peripheral disruption associated with poor metabolic health, which alters reward-related neural systems and via this route may drive depression symptoms. It also constitutes a promising therapeutic target: For instance, exercise improves motivation across a range of psychiatric disorders, including depression (*110*) and is effective in depression with comorbid type 2 diabetes (*111*). Mechanistically, exercise both reduces inflammation and enhances dopamine signaling (*112*, *113*), believed to be key to its therapeutic effects (*114*).

Neuroendocrine disruptions and inflammation are themselves interrelated, including via the effects of interventions: In addition to changing inflammation, exercise also improves peripheral and brain insulin sensitivity (*115*). This highlights the overlap and complexity in the different causal pathways between metabolic and mental health. Nevertheless, neuroendocrine and inflammatory disruptions have convergent effects on reward, learning, and motivation-related neural circuits, suggesting that they influence depression by disrupting mediating neurocognitive mechanisms.

#### Additional pathways: vagal signaling and the gut-brain axis

In the current review, we focus on neuroendocrine signaling and inflammation as candidate mechanisms by which metabolic ill-health may cause psychiatrically relevant changes in neurocognition. However, a plethora of other biological systems likely also play a role. While a full description of all possible mechanisms is not within the scope of the current review, we briefly highlight two additional pathways of interest here.

First, the vagus nerve, part of the autonomic nervous system, plays a key role in eating behavior, communicating metabolic information to the brain (*116*). Animal studies suggest that diet-induced obesity may interfere with vagal signaling (*117*), and preliminary evidence suggests that the same may be true in human obesity (*118*). Critically, the effects of vagal afferents on the brain are mediated by dopaminergic signaling, suggesting relevance to reward and motivation-related cognition and mood disorders (*119*). Stimulation of the vagus nerve is shown to have antidepressant effects, which recent work suggests is mediated by effects on motivation-related cognition (*120*–*123*).

Second, the gut microbiome and its influence on the brain have been of growing interest in recent years (*124*). The composition of the gut microbiome is highly person specific, resulting from complex interactions between the host and environmental factors (*125*). The microbiome plays a role in energy balance, communicated via the gut-brain axis, and alterations in the gut microbiota can both be the consequence of and contribute to the development of metabolic ill-health, including obesity and insulin resistance (*126*, *127*). The microbiome-gut-brain axis is also suggested to play a role in cognitive and reward processes, partly through interactions with mesocorticolimbic pathways (*128*). Moreover, both animal and human studies have linked the gut microbiota to neuropsychiatric conditions, including depression (*129*, *130*).

### The case for reverse causality

Various physiological changes associated with metabolic ill-health, some of which we review here, have direct consequences for depression-relevant neural and cognitive processes. These changes describe one putative path from metabolic ill-health to depression. But it is insufficient (and overly simplistic) to attribute metabolic and mental health comorbidity to metabolic-induced disruptions in neural systems. There is substantial evidence that this path is bidirectional, with compelling longitudinal evidence suggesting the stronger relationship runs from depression to diabetes (*113*). In the following section, we review the mechanisms driving this directional pathway. However, the two should not be taken as competing explanations: It is much more likely that both directions (and common causes of both) contribute to the risk and maintenance of depression and diabetes.

A recent large cohort study (*N* = 672,823) found that psychiatric conditions in early adulthood increased later cardiometabolic risk (*114*). One important contributor is long-term use of psychotropic medications associated with metabolic risks (*18*, *19*). Alternatively or additionally, dietary and lifestyle differences occur in psychiatric disorders like depression, increasing risk for metabolic ill-health (*115*). But why and how? We suggest that this “reverse causal” explanation likely occurs via similar changes in neurocognitive systems, including dopaminergic pathways. As illustrative examples, we outline how known neurocognitive changes in reward- and effort-based decision-making could cause behavioral changes that precipitate metabolic ill-health. Thus, depression could confer metabolic risk by altering neural processing of energy-relevant behaviors such as diet and movement.

#### Reward processing

Changes in reward processing are strongly associated with depression and other psychiatric conditions (*131*). Individuals with depression show impairments on behavioral reward-processing domains, including reward bias [e.g., (*132*)], option valuation [e.g., (*133*)], and reward learning [e.g., (*134*)], as well as abnormalities in reward-related neural processing [e.g., (*135*)]. By altering the cognitive-behavioral responses to food-related rewards, changes in reward processing could alter food-related behavior and confer metabolic risk via dietary changes.

Several studies have linked increased appetite and weight gain in depression to brain alterations, particularly in reward- and interoception-related regions. A group of unmedicated depressed individuals with current hyperphagic symptoms exhibited greater neural responses to food images in regions in the mesolimbic system, including the orbitofrontal cortex, ventral striatum, ventral pallidum, and the putamen compared to body mass index and depression severity-matched hypophagic depressed and healthy controls (*136*). Moreover, hyperphagic depressed participants showed an increased response in the anterior insula, as well as a negative relationship between food pleasantness ratings and neural activity in the left and right dorsal mid-insula (*136*). In a subsequent study, again comparing unmedicated participants with hyper- or hypophagic depression and healthy controls, matched by body mass index and depression severity, hyperphagic depressed participants exhibited a positive relationship between insulin resistance and magnitude of food-cue reaction in the posterior and dorsal mid-insula cortex (*137*). Further, in individuals with hyperphagic depression, inflammatory markers were associated with an increased coupling between food pleasantness ratings and activity in the orbitofrontal cortex and anterior and mid insula but dampened coupling in the posterior insula (*138*). Notably, hyperphagic depression was linked to reduced surface area of the anterior insula (*139*) and attenuated functional connectivity of the NAc to the insula (*140*). Overall, these results suggest that increased appetite in depression may be associated with changes in reward and interoceptive neural circuits.

Neural changes in these reward circuits could suggest a particular disruption in the “wanting” process in depression, which would alter food-related anticipation. This is corroborated by behavioral evidence. Some studies report increased pleasantness ratings to food cues in participants with hyperphagic depression, compared to hypophagic depression (*138*) and healthy controls (*136*). But by collecting repeated rating of food rewards with increasing reward proximity, Schulz and colleagues dissected reward behavior toward food into anticipation and consummation and found differential effects between individual with atypical and melancholic depression: While participants with melancholic depression showed reduced wanting during the anticipatory stage, participants with atypical depression did not (*11*). Instead, participants with atypical depression showed more pronounced increases in wanting as the food reward moved closer, leading to enhanced wanting in atypical depression during the consummatory phase (*11*). Neither group of depressed participants showed deficits in liking as food was consumed, suggesting no hedonic deficits in either subtype of depression. Overall, these results demonstrate that both subtypes of depression show differences in wanting of food rewards, but that the specific reward stage at which this emerges differs by subtype. The increased wanting during consummation seen in atypical depression could contribute to overeating, weight gain, and associated metabolic disruptions specific to this subtype. This suggests atypical depression, in particular, may drive a reverse causal relationship between metabolic and mental health, with general reward processing changes (present in both subtypes) specifically manifesting in consummatory food stages, altering food-related behavior with downstream metabolic consequences.

Dietary differences in depression may also extend to particular macronutrient compositions. Depression and depressed mood have been linked to an increased craving for carbohydrate-rich foods (*141*, *142*). In line with this, an experimental study using food cues found depressed participants compared to healthy controls prefer carbohydrate-rich foods compared to fat- or protein-rich foods (*143*). Notably, this enhanced carbohydrate preference was associated with depression severity, anhedonia, and anxiety symptoms. Overall, altered processing of food rewards in depression may lead to different behavioral responses to food, which has not only acute effects on eating behavior but also long-term metabolic consequences.

#### Effort-based decisions

A similar pathway could occur via effort-related decisions, conferring metabolic risk via altered movement behavior. The association between psychiatric disorders and disrupted effort-based decision-making is a widely replicated finding. Patients with major depression [e.g., (*144*)] and people with heightened transdiagnostic symptoms of apathy [e.g., (*145*)] and anhedonia [e.g., (*146*)] show altered effort-based decision-making, such that they are less willing to exert effort for rewards.

These disruptions in effort-based decision-making indicate a shift away from behavior that expends energy and toward behavior that conserves energy. One way that this can manifest is a change in everyday lifestyle, particularly elements that increase risk for metabolic ill-health. For instance, more pronounced effort discounting has been linked to a more sedentary lifestyle (*147*) and worse adherence to a weight-loss treatment (*148*). Over time, this cognitive shift and subsequent behavioral changes conserving energy could reasonably increase the likelihood of metabolic ill-health via changes in muscle mass, muscle-to-fat ratios, and other physiological precipitants (*149*).

Reward- and effort-based decision-making comprises two well-replicated neurocognitive changes in depression, both of which could confer risk of metabolic ill-health through behavioral change. Equally, as outlined earlier, changes in metabolic health, particularly neuroendocrine signaling and inflammation, could alter dopaminergic function in the brain. These metabolic-to-mental health changes could occur as a consequence of mood changes or preceding mood changes but, in either case, could serve to augment cognitive vulnerabilities to depression. Alternatively or additionally, because individual differences in these and related neurocognitive measures exist across the population, cognitive vulnerabilities could confer common risk for metabolic and mental health.

### A common cognitive phenotype?

The final putative causal pathway that we explore proposes that shared cognitive mechanisms or cognitive phenotypes could simultaneously confer risk for metabolic ill-health and psychiatric disorders. In line with this idea, evidence suggests that metabolic and mental ill-health are independently associated with disruptions in motivated behavior. Our recent findings suggest significant differences in effort-based decision-making between individuals with type 2 diabetes and healthy controls, despite no differences in psychiatric symptoms (*150*). Notably, computational modeling identified the same parameter driving motivational differences in type 2 diabetic participants as previously found in relation to psychiatric symptoms (*151*). However, in patients with major depressive disorder who are overweight or obese, there is also an additional contribution of worse metabolic health metrics to blunted effort-based decision-making, even after accounting for severity of depression (*152*). While neither of these studies tests causal relationships, together, they suggest parallel, separable contributions of depression and metabolic ill-health on effort-based decision-making. However, future studies are needed to investigate whether these phenotypically parallel differences in effort-based decision-making stem from shared or distinct biological mechanisms. The development of more biologically plausible computational models, for instance, integrating the agent’s internal state, may be of particular importance when tackling this question.

Convergent cognitive phenotypes conferring risk for both could originate from shared genetic, behavioral, and environmental exposures. We will briefly describe examples for such “common risk” pathways. First, both depression (*153*) and metabolic health (*154*) have genetic contributions, including substantial shared genetic components (*155*). Intriguingly, a systematic review of the genetic overlap between mood disorders and cardiometabolic disease found an overrepresentation of genes encoding for molecules involved in dopaminergic neurotransmission (*156*), which could manifest as cognitive differences in reward processing. Further, the TaqIA polymorphism, commonly studied in the context of addiction, has been linked to both altered striatal dopamine function and metabolic energy regulation (*157*).

Additionally, both mental and metabolic health conditions are closely linked to disruptions of sleep-wake patterns. Shift work, for instance, is closely linked to metabolic disruption (*158*) and increased risk for depression (*159*). Through irregular sleep-wake timings and decoupling from our daily environmental rhythms, shift work disrupts the circadian rhythm (*160*). The circadian rhythm is closely linked to dopamine signaling. Animal studies have demonstrated disrupted dopamine signaling following knockout of circadian genes (*161*). In humans, dopamine availability is linked to circadian gene mutations (*162*) and behavioral circadian rhythm proxies (*163*). Circadian misalignment is associated with reduced medial prefrontal cortex and striatal reward reactivity (*164*). Recent work from our lab has found that individual differences in circadian rhythm interact with the time of day to affect effort-based decision-making, disrupting effort-based decision-making at chronotype-incompatible times (*151*). In sum, circadian disruption, for instance, through shift work, may convey both psychiatric and metabolic risk by altering dopaminergic signaling and, consequently, reward processing and behavior.

Diet composition may also represent a common risk factor for both metabolic dysfunction and depression, possibly mediated by neurocognition. Animal studies have consistently shown that a high fat diet can impair metabolic health (*165*, *166*), disrupt dopaminergic signaling (*167*, *168*), and cause depression-like behavior (*169*–*171*), even in the absence of weight gain (*117*). Similarly, human studies show that an increased intake of saturated fat can lead to weight-independent visceral adipose fat accumulation (*172*), as well as alterations to the neural response related to both food and nonfood rewards (*173*). Last, reports of positive correlations of depressive symptoms with dietary intake of saturated fats (*174*) and circulating saturated fatty acid palmitate (*175*) in humans further suggest a link to mental well-being.

Third, mental and metabolic health are both associated with exposure to environmental stressors, including adversity (*176*), stress (*177*), and inflammation. As described above, inflammation can be a consequence of metabolic disruption (*65*) and has, hence, been discussed as a pathway by which type 2 diabetes can change reward processing and, consequently, increase psychiatric risk (*106*). However, there are additional possible directions of causality; inflammation (from other causes) is known to worsen insulin resistance (*178*). Hence, raised levels of inflammation, for instance, due to viral infections (*179*) or environmental pollution (*180*) could worsen both mental and physical health, including (through not limited to) depression and diabetes.

### Interim conclusion

It is unlikely that any of the described causal pathways operate in isolation, even within a single individual. Neural changes originating from disturbances in metabolic signals could plausibly worsen mental health (i.e., the metabolic-to-mental health causal pathway). But the same neural changes could also affect behavior, which could worsen metabolic health. In the other causal direction (i.e., from mental to metabolic health), while mental health–related cognitive changes could change behavior leading to metabolic ill-health, any consequent metabolic disruptions would, nevertheless, affect neural insulin signaling, with consequences for cognition and mental health. Both causal directions could also be amplified by preexisting common factors, whether genetic, behavioral (e.g., shift work), or environmental (e.g., inflammation resulting from pollution).

**Fig. 1.**
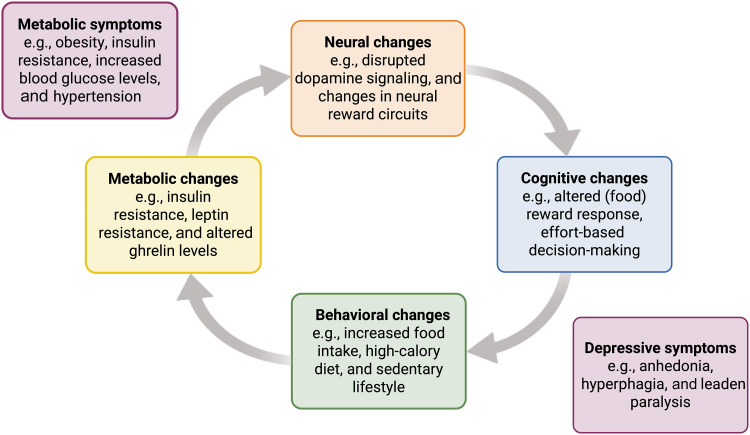
A vicious cycle between neurocognition, behavior, and metabolism. Neural changes, like disrupted dopamine signaling, can lead to cognitive changes, like reduced reward responsiveness, which may translate to changes in behavior, such as increased food intake. These behavioral changes can affect metabolic health, for instance, implicating insulin sensitivity, which, in turn, can affect neural processing, feeding back into the cycle. Together, these processes can give rise to both metabolic ill-health and depressive symptoms. Created in BioRender. S. Mehrhof (2025), https://BioRender.com/ty75wcf.

Rather than viewing the possible causal pathways described in the above sections as distinct effects, a more plausible explanation is that there is mutual influence of metabolic and mental health via cognitive and behavioral changes. Specifically, cyclical causal pathways between cognition, energy-metabolism, and behavior could give rise to and maintain the comorbidity between metabolic ill-health and mental health conditions like depression (Fig. 1). The closed-loop nature of these suggested pathways between neurocognition, behavior, and energy metabolism, wherein changes at one level have consequences at other levels, however, comes with the challenge of identifying the origin of the vicious cycle (*24*). There are likely multiple points of entry to this cycle, and different patients with the same metabolic-psychiatric comorbidity may exhibit diverse contributions of the different pathways involved. An open question remains as to the specific causal factors relevant to individual patients, which might be relevant for preventative interventions: This would reveal which point in the cycle disruption is possible and desirable for each person. Computational modeling may offer a way of disentangling the contribution of cognitive pathways in an individual, possibly leading to more targeted and personalized interventions across treatment domains (e.g., cognitive behavioral therapy in conjunction with pharmacological treatments targeting metabolic dysfunction).

## THE INTEROCEPTIVE SYSTEM AND ITS ROLE IN ALLOSTASIS

**Fig. 2.**
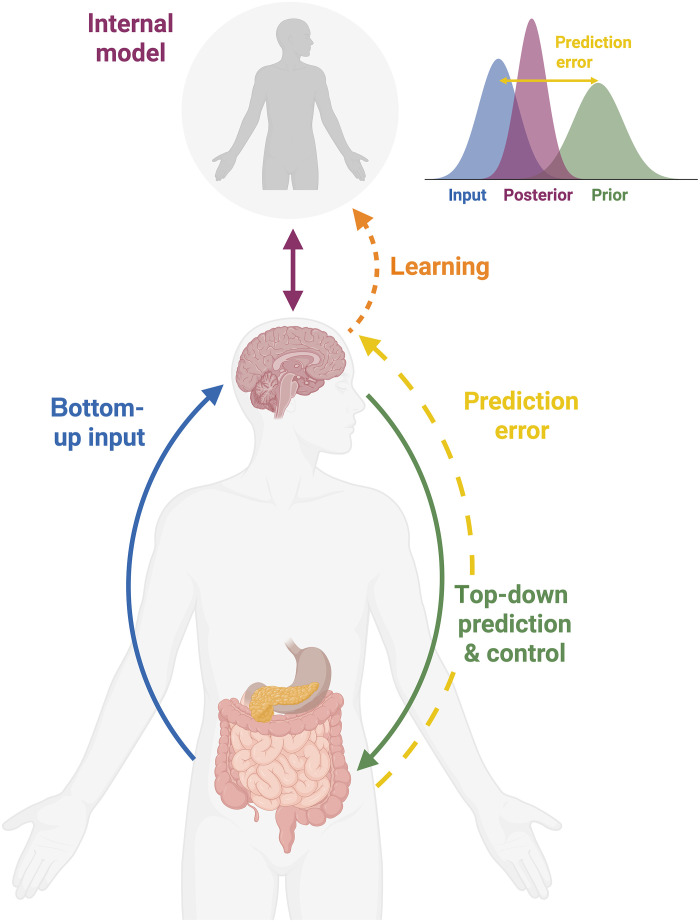
The interoceptive allostatic system. Interoceptive signals form bottom-up signals that are integrated with exteroceptive information and prior experience to inform the brain’s internal model of the body in its environment. Based on this internal model, the brain predicts bodily signals and prospectively regulates physiological processes, ensuring stability through change. Discrepancies between model predicted and actual (bottom-up) bodily signals lead to a prediction error, which can be used to refine the internal model through learning. As illustrated in the plot in the upper right corner, learning can be understood as the updating from prior to posterior beliefs. The extent of learning depends not only on the prediction error but also on the relative precisions (width) of the input and prior distributions: The more precise (narrower) the input distribution and the weaker (wider) the prior, the more the posterior belief will be able to shift away from the prior, reflecting greater learning. Note: The selection of organs depicted here is for illustrative purposes only and reflect the focus on energy metabolism. Created in BioRender. S. Mehrhof (2025), https://BioRender.com/zvs5rsf.

Signals from the body have the capacity to alter neural circuits relevant to mental health, and neurocognitive disruptions present in mental health can alter physiology via behavior; common causes might engender both. But in all cases, whether originating in bodily disruption or neurocognitive changes, these alterations interact with the brain’s interoceptive allostatic system (Fig. 2).

Interoception is a key process in maintaining physical and mental health. Interoceptive information includes signals about the body’s state, essential to monitor its need for water, glucose, oxygen, etc. Interoceptive information is processed hierarchically and integrated with environmental cues and prior knowledge to build an internal model of the body. Using this internal model, the brain can infer potential consequences of an internal state and adjust accordingly. Hence, rather than waiting for an imbalance to occur, the brain predicts future needs and prepares to satisfy them before they arise (*19*, *22*, *181*–*184*). This predictive regulation of future physiological needs is termed allostasis: anticipatory regulation of bodily state.

Nearly all interoceptive research focuses on signals originating in a specific organ of the body. Most studies investigate cardiac interoception (e.g., the sensing of heartbeats or the influence of heartbeats on cognition and the brain) and respiratory interoception (e.g., the sensing of breath restriction or the influence of breathing restriction on cognition and the brain). A smaller number investigate gastric interoception, for example, by probing fullness sensations in the stomach [with a water load challenge; (*185*)] or, more recently, vibrations in the stomach [with an ingestible mechanical pill; (*186*)]. This research overwhelmingly suggests that disrupted interoception plays an important role in psychiatric disorders, through alterations in signaling, perception, prediction, or some combination thereof (*21*, *27*, *28*). Intriguingly, disrupted interoception may also represent a link between physical health conditions and mental health (*27*). For instance, asthma shows a high comorbidity with anxiety disorder (*187*), and both asthma (*188*) and anxiety (*189*) are characterized by reduced respiratory interoceptive accuracy.

Maintaining interoceptive allostasis is critical across all systems, with general disruptions in allostasis cited as a framework for brain disorders more generally (*190*). But sources of interoceptive information are much more diverse than those originating from single organs: Immune, endocrine, blood osmolality, and metabolic signals (among others) constitute less well-researched components of the interoceptive milieu.

A disruption in interoceptive information processing, therefore, would necessarily include phenomena such as metabolic information and provide a link between both possible directions of causality for metabolic-mental health influences. A change in predicted metabolic states would have consequences for all closely interconnected aspects of cognition, including motivation and reward processing. Across the following two sections, we first introduce interoceptive allostasis as a central mechanism of metabolic energy regulation, tightly linked to neurocognition. Next, we propose that disruption of interoceptive energy allostasis may promote and maintain comorbidity between metabolic and mental health conditions, potentially driven by altered detection, prediction or learning of internal energetic states.

### Interoceptive allostasis to support energy balance

Interoceptive allostasis seems critical for metabolic information. Energy balance is a crucial function to regulate prospectively: Minimizing the risk of energy scarcity in the face of any disruptions maximizes an organism’s chances of survival. Its criticality is indirectly supported by neural and humoral pathways supporting energy regulation, which are largely evolutionarily conserved: hormonal and neural feedback from the gut to the brain regulate feeding behavior in drosophila, lampreys, and blowflies (*191*).

Interoceptive signals of energy supply and need are largely derived from the gut and communicated to the brain via various mechanisms. For instance, endocrine signals like ghrelin, insulin, or leptin reach the central nervous system through blood circulation, while various forms of gut sensation (chemosensory and mechanosensory) communicate nutrient information to the brain via vagal sensory neurons (*191*, *192*). Different metabolic interoceptive signals operate on different timescales, allowing communication of both short- and long-term shifts in energy status. For instance, after food intake, vagal stretch and tension sensors signal the amount of nutrients temporarily stored in the gastrointestinal tracts, providing an estimate of the energy that will soon become available through metabolism (*192*). In contrast, leptin communicates long-term signals of peripheral energy state (i.e., amount of energy now stored as fat in the body) (*57*, *58*).

Together, these metabolic signals form the “bottom-up” energetic input that is integrated in the brain, particularly in the nucleus tractus solitarus, hippocampus, hypothalamus, insula, and basolateral amygdala that regulate satiety and feeding behavior (*191*, *193*–*195*). However, these afferent metabolic signals are noisy and do not regulate energy balance in a reflexive or direct manner; rather, interoceptive metabolic signals must be detected, interpreted, and integrated. Within the allostatic framework, this means that the incoming interoceptive information is integrated with exteroceptive information and prior experience to form the brain’s internal model of the self in the environment.

To illustrate the centrality of metabolic bottom-up signals in energy allostasis, consider the reinforcing value of food. Primary reward signals associated with food are not generated during consumption but, rather, are of interoceptive origin, generated postorally during digestion and absorption (*196*). In rodents, for instance, dopamine release in response to sugar consumption can be blocked by the coadministration of an antimetabolic agent (*197*). In humans, the striatal response to flavor cues has been shown to reflect the metabolic response learned through prior association with calories (*198*, *199*). In turn, the metabolic response to calories can be influences by the matching of sweet taste and caloric load (*198*). This postoral reinforcement is advantageous, allowing the coupling of nutrient consumption with behavior, reinforcing the selection of nutrient-rich foods in future. Conversely, postoral malaise signals powerfully reinforce avoidance of potentially toxic foods. Animals learn to associate novel flavors with delayed gastrointestinal signals of malaise after only a single learning episode (*200*). This occurs via reactivation of a specific population of amygdala neurons which enable association of postoral malaise with oral (or potentially olfactory and other preoral) signals, causing future avoidance of the novel flavor (*200*).

While metabolic signals provide insight to the brain about the body’s current metabolic state, the brain also makes predictions about novel, incoming metabolic signals. These top-down predictions are compared to the bottom-up signals; a discrepancy between the two leads to a prediction error. Prediction errors underpin learning: To minimize the error signals, the internal model is recalibrated, allowing more accurate future predictions. In this learning process, precision estimates of the prediction error can be used to infer relevance and reliability of the signal, guiding the extent of learning.

Model predictions and prediction errors are also used for top-down regulation of energy balance. For instance, the brain can exert prospective control over metabolic energy balance by inhibiting the activity of hunger neurons in response to food-predicting cues, before actual consumption (*201*, *202*). Alternatively, central control over energy balance can be exerted via higher-order effects on cognitive processes. A well-replicated effect from the animal literature is the motivating effect of hunger (*203*–*205*); this effect is also seen in lean humans (but is absent in obese humans) (*76*).

Interoceptive allostasis provides a framework to explain how metabolic predictions, on short and long timescales, influence cognition, guiding behavior in line with anticipated energetic needs. Prediction errors computed from the difference between expectations and bottom-up signals (modulated by their respective precision) induce learning, calibrating expectations for better future prediction. This energy allostasis enables prospective regulation of metabolic state, allowing us to adapt to a changing energy landscape to maximize chances of survival.

### A role for disrupted energy allostasis in driving mental-metabolic health comorbidity

**Fig. 3.**
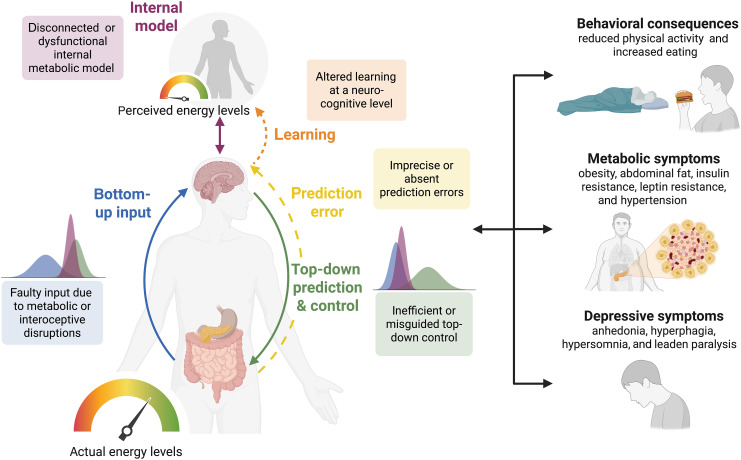
Energy dysregulation in the interoceptive allostatic system. Interoceptive energy allostasis can be brought out of balance via various disruptions at all levels of the system, examples of which are visualized here. Dysfunctional energy regulation can result in a mismatch between the actual and perceived energy levels, such that energy levels are systematically underestimated. Disrupted energy allostasis has consequences regarding behavior, metabolic health, and depressive symptoms, which feed back into the dysfunctional system. Created in BioRender. S. Mehrhof (2025), https://BioRender.com/1hrd03i.

Successful energy allostasis requires coordination between bodily systems, reliable prediction of energy needs, effective regulation of internal states, and dynamic adaptation to changing demands. But what happens when this intricate system is brought out of balance? While the regulatory mechanisms of interoceptive allostasis ensure energy balance through a virtuous cycle, ensuring stability through change, disruption of the system can lead to a vicious cycle of energy dysregulation (Fig. 3). Disrupted energy allostasis can originate in changes to metabolic signals (e.g., faulty sensory input, causing faulty prediction errors), neural interoceptive changes (e.g., faulty predictions of energetic needs), or areas of interaction between the two, such as altered learning from metabolic signals. Yet, due to their centrality to energy allostasis, each of these changes can induce a system-wide disruption with behavioral, metabolic, and psychiatric consequences.

We propose that dysregulation in interoceptive energy allostasis generates and maintains comorbidity between metabolic ill-health and mental health conditions like depression. Dysregulation in energy allostasis can follow from both mental and metabolic pathologies, as well as give rise to them. As we outline below, cognitive and metabolic alterations present in depression and metabolic ill-health serve as insults to the interoceptive allostatic system, with the capacity to trigger, maintain, and aggravate disrupted energy regulation. At the same time, there are behavioral, metabolic, and psychiatric consequences of dysregulated energy allostasis: reduced physical activity and increased eating, metabolic disruptions like insulin resistance, and motivational deficits, like apathy and anhedonia.

There are numerous potential origins of disrupted allostatic energy regulation. For instance, adversity such as living in poverty or an unsafe environment, experiencing social disparities, traumatic events, or chronic stress are thought to cause an “allostatic overload,” leading to system-wide dysregulation across various bodily and psychological systems, including (but not limited to) energetic regulation (*190*). Our proposed framework focuses specifically on pathways by which changes in metabolic and/or cognition may disrupt allostatic energy regulation (irrespective of distal origin). This enables us to make specific predictions regarding the origins of comorbidity between metabolic ill-health and psychiatric conditions, particularly major depression, key sources of current health burdens. Over the next sections, we provide a nonexhaustive list of insults to different parts of the interoceptive allostatic system and the role that we hypothesize that they play in driving mental and metabolic health conditions.

#### Faulty metabolic signals

Hormones such as ghrelin, leptin, and insulin play key roles in metabolic regulation, communicating energy-related state information from the periphery to the brain (*191*, *192*). But when these signals become unreliable, for instance, due to insulin resistance or reduced leptin sensitivity, the precision and reliability of sensory information deteriorate. These faulty metabolic signals would then alter interoceptive energy allostasis from the bottom up, by altering the precision estimates of signals. Mathematically, reduced precision of metabolic signals would lead to increased discounting of incoming energetic information (as shown in the bottom-up input plot in Fig. 3), reducing learning from any metabolic prediction errors, and increasing reliance on (potentially inaccurate) metabolic predictions.

Sources of disruptions to bottom-up metabolic signals include longer-term metabolic conditions like obesity, in which blunted neuronal responses to nutritional signals have been reported (*118*). However, even short-term changes in lifestyle could contribute to disrupted bottom-up signaling of energy signals. For instance, a short-term change in diet can disrupt insulin action in the brain (*51*), and only 4 days of shift work can significantly reduce peripheral insulin sensitivity (*206*). Therefore, at multiple timescales, faulty metabolic signals have the capacity to dysregulate energy allostasis, altering the brain’s ability to accurately detect and predict changes in metabolic need. Changes in these neural functions would necessarily result in behavioral alterations (e.g., increased energy conservation) as well as symptoms related to perceived energy levels (e.g., apathy and anhedonia).

#### Disruptions to metabolic interoception

Even when bottom-up metabolic signals remain intact, disruptions in metabolic interoception could interfere with their processing. This would lead to reduced or faulty integration into one’s internal model of energy needs and regulation. Depending on the nature and directionality of the interoceptive disruption, this could result in an allostatic system hypo- or hyperresponsive to metabolic signal changes.

Metabolic interoceptive disruptions may occur at various interoceptive levels (*19*), including interoceptive accuracy (the ability to detect changes in energy levels, such as accurate hunger perception), interoceptive attention (the capacity for changes in energetic signals to capture attention), and attribution (for instance, the valence of energetic signals, how unpleasant hunger and fullness feel). Numerous studies have shown disruptions in interoceptive accuracy in mental health conditions, including cardiac and respiratory interoception [as reviewed elsewhere (*27*)]. Far fewer studies examine metabolic interoception (at any level). Nevertheless, emerging data tracking mood, hunger, and accuracy of metabolic interoception suggest that individuals with high metabolic interoceptive accuracy have fewer fluctuations in mood ratings (*207*). Critically, in this study, the effect of glucose levels on mood (i.e., low blood glucose worsening mood) was fully mediated by someone’s awareness of their own hunger, i.e., the accuracy of their metabolic interoception. Changes to metabolic interoception [for example, in the context of general interoceptive dysfunction in mental health conditions; (*21*)] could plausibly have consequences for mood as well as energy-related behaviors.

#### A disconnected internal metabolic model—From prediction to control

The brain’s internal model of the body dynamically integrates new sensory input and prior expectations. However, when there is an over- or underreliance on one of these sources, the model can become dysfunctional. The result is an internal model that is disconnected from current sensory input or biased toward certain states, such as chronic energy deficiency. An internal metabolic model may become disconnected from sensory input due to faulty afferent metabolic signals or disruption to metabolic interoception, as described in the sections above. Alternatively, the model may become “stuck” in a state that was adaptive in a previous context but maladaptive in the present, due to insufficient updating. This could result from learning deficits, where prediction errors fail to adjust the model as they should (as outlined below). In both cases, a dysfunctional model is characterized by faulty predictions (or priors) for its current state, which has critical consequences for the maintenance of allostasis for prediction and control of bodily states.

Our internal model is used to generate predictions about future sensory inputs. When the model is inaccurate, predictions become imprecise or incorrect, which we hypothesize can undermine allostasis. First, it can lead to prediction errors, mismatches between the model-predicted and actual signals, which can become persistent and imprecise, as described below. Second, inaccurate predictions can impair top-down regulation of bodily processes. This could result in weak or inefficient control (due to reduced prediction precision; see Fig. 3, top-down prediction plot) or in actively misguided regulation. A concrete, computational example for such misguided control comes from effects on higher-order cognition, including effort-based decision-making or reward sensitivity: Inaccurate model predictions of chronic metabolic energy scarcity could lead the brain to downregulate energy expenditure by increasing effort costs during computations of effort-based decision-making. Clinically, these changes in motivational cognition correspond to amotivational symptoms, including anhedonia, apathy, and anergia. Alternatively or additionally, a model-predicted state of energy scarcity could trigger increased sensitivity to food rewards, with consequences for metabolism-relevant behaviors (e.g., diet).

However, even when the internal model is accurate, top-down control may fail if regulatory mechanisms are compromised. For instance, the internal model may correctly predict food ingestion and initiate insulin release, but insulin resistance would render this regulatory action less effective, making the internal model’s control faulty in that state. Similarly, deficits in higher-order cognitive mechanisms due to psychiatric conditions may render energy regulation through behavioral adaptation less effective in the first place. An internal metabolic model has the capacity to adapt to changes in the external or internal environments. But a failure to adapt puts a model at risk of disconnection, leading to faulty predictions and ineffective control.

#### Imprecise or absent prediction errors

A key source of information to precipitate updating an internal metabolic model and ensuring future predictions and control are prediction errors. However, learning from prediction errors is metabolically costly; it necessitates the encoding and consolidation of information, brain computations associated with an energy cost (*22*, *208*, *209*). Because of this, the brain must estimate their precision to determine whether they are worth integrating. If a prediction error is estimated to have low precision—perhaps stemming from faulty sensory input, interoceptive processing deficits, or a poorly calibrated internal model—then it may be discounted or ignored altogether. This leads to a failure in learning, leaving the internal model increasingly out of sync with reality and worsening energy dysregulation (*210*).

Just like a failure to learn from prediction errors can impede allostasis, so can a lack of prediction errors. Depression can lead someone to withdraw from daily life and activity. This is particularly pronounced for energy-relevant behaviors: Depression is marked by changes to appetite, sleep, sex drive, and movement. Interaction with new outcomes, driven by exploratory behaviors, is necessary to generate prediction errors and correct faulty internal models. Without engagement with the environment one can get trapped in outdated or maladaptive models as no prediction errors are generated that may help recalibrate the model (*22*).

#### Altered learning

Last, we suggest that disruptions to learning mechanisms at the neurocognitive level may extend to the metabolic domain, impairing energy allostasis. Differences in reward sensitivity, learning rates, and error processing, well-documented in various psychiatric populations (*131*), may hinder the updating of predictive models. For example, an individual with reduced sensitivity to interoceptive reward signals may fail to learn appropriate associations between food intake and energy states. This change in learning could have further downstream effects, thereby shaping cognition and behavior long-term and play a key role in sustaining maladaptive allostasis and energy regulation.

What we eat also influences metabolic learning specifically. Taste, caloric load, and the (mis)match between the two influence the neural and metabolic response to food consumption, via metabolic learning mechanisms, which adaptively function to predict the energetic value of flavors (*198*). A diet rich in flavor-nutrient mismatched foods [e.g., sugar-free sodas and ultra-processed foods; (*211*)] could, therefore, also disrupt metabolic learning, leading to impaired interoceptive accuracy and dysregulated energy balance.

## FROM MODEL PREDICTIONS TO CLINICAL TRANSLATION

Our interoceptive model for energy allostasis provides a conceptual model explaining the mutual drivers of poor metabolic and mental health, a potential major contributor to the high comorbidity between depression and diabetes. From this model, it becomes evident that the different trajectories of disrupted energy allostasis suggested here are inherently interconnected and mutually reinforcing. Likewise, the behavioral, metabolic, and psychiatric consequences that arise from the dysfunctional model may feed back into the cycle via their effects on metabolic signals, neural processing, or both.

There are specific predictions arising from this model that provide fruitful ground for future investigations. If entry points exist at multiple levels leading to metabolic-mental health comorbidity, so too might potential interventions (which may be distinct from an individual’s point of entry). Based on our model, we would, for instance, hypothesize that patients with metabolic signal disruptions (e.g., diabetes) could benefit from interventions at the interoceptive level such as interoceptive training (*212*), in addition to existing treatments targeting the signal (e.g., insulin sensitizing drugs like metformin). We would predict that an interoceptive intervention might reduce the risk of mental health comorbidity by improving the accurate integration of metabolic information to the brain, thus alleviating cascading consequences for cognition and behavior, described in our model. Our model would also predict that the risk of depression would be highest in patients with less well-treated diabetes. This is because noisier metabolic signals, as can be expected in poorly controlled diabetes, would correspond to more rapid and severe deficits in interoceptive sensing of metabolic state, in turn, leading to broader disruptions in energy allostasis. In psychiatric patients, we would predict that our model of dysfunctional energy allostasis would most likely apply to patients with a symptom profile characterized by reduced motivation, reduced physical activity, hypersomnia, and increased appetite. Hence, this subgroup of patients may most benefit from metabolic interventions to treat psychiatric symptoms, a category that could include GLP-1 agonists (*213*), while also being at increased risk for metabolic comorbidity, warranting consideration of preventative interventions. Together, these provide testable predictions as well as potential avenues for pharmacological and psychological interventions that could target metabolic-depression comorbidity.

Last, our model would predict that general changes in reward learning apparent in depression could cause deficits in forming and updating expectations about the nutritive properties of food (i.e., metabolic learning), which could lead to common appetite disruption symptoms like hyperphagia. In future work, metabolic learning could serve as an entry point for putative interventions targeting metabolic health in patients with depression.

To test and, potentially, falsify the model of interoceptive energy allostasis, rigorous empirical designs will be needed (see Box 1). Intervention studies are key to establishing the putative causal roles of each level of the system. On one hand, causal interventions targeting bottom-up signals exist and are ripe for further testing (*45*, *214*). In contrast, interventions directly targeting allostatic prediction remain scarce. Nevertheless, one way forward could involve experimental manipulation of prior beliefs about bodily states and their reliability (as in the context of nicotine beliefs) or, in future, targeting neural regions associated with allostatic predictions via brain stimulation, with any putative interventions validated by comprehensive testing of interoceptive levels (*215*, *216*). Moreover, to harness the potential of the proposed model in furthering research and clinical work, outstanding questions will need to be addressed, including, for instance, temporal dynamics of interoceptive allostasis (and disruption thereof), the role of resilience, and explanatory value for other pathologies (Box 2).

Box 1. **Toward empirical tests of interoceptive energy allostasis.**

Our model of interceptive energy allostasis makes a number of predictions about the development and maintenance of comorbidity between mental and metabolic ill-health. Some of these predictions are readily testable; others will involve methodological innovation. Below, we outline five promising avenues to probe this framework empirically.

1) **Experimental pharmacology targeting bottom-up metabolic signals.** Of particular interest may be intranasal insulin to probe insulin’s effect on the brain, with only minimal peripheral effects (*45*). However, other metabolic hormones could also be targeted, for instance, through intravenous infusions [e.g., ghrelin infusion; (*214*)].

2) **Emerging technologies to trigger endogenous physiological signals such as ingestible bioelectronic capsules.** These offer another approach of manipulating afferent metabolic signals. Although now only tested in animal models (swine), different ingestible bioelectronic capsules have been developed and demonstrated to stimulate endogenous metabolic hormones release [ghrelin and GLP-1; (*217*)], as well as stimulate gastric mechanoreceptors, thereby influencing satiety (*218*).

3) **Experimental paradigms targeting allostatic prediction and control.** Direct manipulation of prior expectations (or their precision) could allow us to experimentally test the effect of beliefs on allostatic prediction and control, as has been shown in the context of nicotine consumption (*216*). Combining such interventional design with novel paradigms distinguishing top-down and bottom-up interoceptive processes (*215*) may offer valuable insight to individual differences in allostatic prediction and control.

4) **Flavor-nutrient conditioning paradigms, which offer a direct window into interoceptive learning.** Study designs could include assessing individual differences in flavor-nutrient learning and relating them to metabolic or psychiatric health indices or cognitive phenotypes, as well as assessing changes in flavor-nutrient learning as one undergoes an intervention targeting metabolic health (e.g., GLP-1–based treatment), psychiatric health (e.g., cognitive behavioral therapy), or interoception (e.g., interoceptive training).

5) **Computational modeling.** Last, a way forward in studying brain-body interactions is the explicit integration of an agent’s biological state, its internal metabolic and interoceptive milieu, into computational models of cognitive processes (*196*). This shift moves beyond treating variables like reward and effort as abstract constructs, instead conceptualizing them as inherently dependent on the agent’s current physiological state and goals. Moreover, computational models incorporating physiological states would allow us to formalize hypothesized mechanistic relationships between physiology and cognition, as well as empirically test them.

Box 2. **Outstanding questions.**

1) How are different temporal dynamics integrated in the interoceptive allostatic system, for example, how are fast-changing insulin levels integrated with the slow-changing input of leptin levels to estimate the body’s current energy state? Or, how is fast-acting control through up- and down-regulation of insulin secretion coordinated with slower-acting regulation through cognitive mechanisms? Last, how are transient energetic states such as hunger after an overnight fast differentiated from more enduring states such as starvation due to impoverishment?

2) What methods could differentiate between disrupted metabolic bottom-up signaling and disrupted interoception of metabolic bottom-up signals?

3) Do individual differences in neurocognitive processes—reward, learning, and effort—in exteroceptive contexts extend to metabolic interoceptive contexts? For example, do learning differences in depression translate to altered learning from afferent metabolic signals?

4) Do interoceptive deficits reported in psychiatric populations extend to signals of current energy state?

5) Could disrupted interoceptive energy allostasis also have explanatory value for other energy-related psychopathologies, such as eating disorders?

6) Do effective metabolic and psychiatric treatments alter disrupted energy allostasis?

7) Do developmental periods associated with marked changes in energy-related physiology—puberty, pregnancy, breast feeding, and menopause—alter interoceptive allostasis? Can our framework offer insights to the increased risk for the onset psychiatric disorder seen in those developmental windows? Can we identify risk and protective factors for the onset of metabolic and psychiatric ill-health during these developmental windows?

8) What can resilience against comorbidity tell us about the causal relationships linking mental and metabolic health? Can we identify individual differences in interoception, neurocognition, allostatic control, or metabolism that explain why some psychiatric patients develop metabolic comorbidity while others do not? And vice versa: Why some, but not all, patients with metabolic disorders develop psychiatric comorbidity?

Understanding the origins of comorbidity between depression and diabetes requires integrating information across levels of explanation: environmental, biological, and behavioral. We review causal routes originating in metabolic health changes, leading to depression, and those originating in the neural mechanisms accompanying depression, leading to metabolic ill-health, as well as common etiological factors across both types of disorders. We focus primarily on depression but throughout discuss mechanisms that are transdiagnostic, applicable to energy-related symptoms across psychiatric disorders (and that differ across subtypes of depression); therefore, we would urge readers to consider this a model of energy-related symptoms, rather than any specific diagnosis.

We next propose that these interlinked pathways form part of a larger interoceptive system driving metabolic-mental health comorbidity. Crucially, this model involves not only direct influences of metabolic state on mental health but also a critical interaction with the brain’s predictive internal model of the body via allostasis. This has repercussions for the conceptualization of comorbidity between depression and type 2 diabetes: Rather than two distinct disorders with mutual risk for one another, we suggest that both form part of a larger system conferring metabolic-mental health risk via changes to energy allostasis, with multiple points of entry. This conceptual shift toward energy allostasis highlights new ways of addressing the challenge of comorbidity, involving working across levels to develop integrative physical-mental health interventions. The mortality gap has long been accepted as a sad consequence of depression. Our interoceptive model of energy allostasis provides an explanation of how comorbidity might occur and propagate and might illuminate ways to break its cycle by targeting underlying mechanisms, offering new avenues to help close the mortality gap.
